# Editorial: Postural Balance Control in Sport and Exercise

**DOI:** 10.3389/fphys.2022.961442

**Published:** 2022-07-12

**Authors:** Giuseppe Marcolin, Matej Supej, Thierry Paillard

**Affiliations:** ^1^ Department of Biomedical Sciences, School of Medicine and Surgery, University of Padua, Padua, Italy; ^2^ Faculty of Sports, University of Ljubljana, Ljubljana, Slovenia; ^3^ EA4445 Laboratoire Mouvement, Equilibre, Performance, Santé (MEPS), Tarbes, France

**Keywords:** balance, posture, control mechanisms, assessment, performance

Postural balance control enables maintenance, achievement, or restoration of a state of balance in any posture or activity. It is, therefore, a determinant in achieving goals in daily life, increasing the quality of life, and maximizing performance in recreational and professional sports. Indeed, in a sports context, balance is one of the performance-limiting factors, and no sport-technique gesture can be efficiently achieved without an effective postural balance control. In sports activities, balance control can also be associated with injury risk. Postural balance control is governed by automatic processes in which the individual is unaware of the adjustment of postural muscle tone and by cognitive processes in which the information about the individual’s own body and the localization of objects in extrapersonal space are required. The complexity of these mechanisms makes postural balance control assessment with a rational approach challenging. In sports and exercise, the assessment of static postural balance provides objective data based on conventional techniques (e.g., measurements of centre of pressure trajectory). However, among athletes or healthy active people, this condition may not be adequately challenging. Conversely, functional and ecological tests provide information on postural ability but may lack objectivity in scoring. Moreover, the assessment of postural balance can be influenced by the postural conditions adopted. For instance, balance performance can change when assessed in a sport-specific context rather than under context-independent postural conditions. Hence, the study of the influence of postural balance control on sports performance or injury risk should necessarily include assessments under dynamic and environmental conditions besides assessments under static and decontextualized conditions. Taking together these considerations, the present Research Topic explored the role of static and dynamic postural balance control on sport and exercise performance, focusing on both static and dynamic assessment methods.

In this Research Topic, three studies addressed the effects of fatigue on postural balance performance. Kozinc et al. investigated the effects of whole-body fatigue on postural sway in single-leg stance and its transient characteristics. They showed that the fatiguing protocol did not increase postural sway, although it tended to be more variable across the trial. Interestingly, postural sway was less affected in females than in males. Marcolin et al. aimed to investigate in male adults whether a fatigue protocol on calf muscle could affect muscle activation strategies and dynamic balance performance over an oscillating platform. They found that the reduction of the electromyographical activity of the soleus during the dynamic balance task after fatigue did not affect the global dynamic postural balance performance, likely due to an overall increase in the calf muscles stiffness. Camargo da Silva et al. extended knowledge of the mechanisms underlying the interplay between fatigue and postural performance associated with the neuromuscular adaptations induced by sports practice. Specifically, they compared gymnasts and age-matched non-gymnasts and showed that fatigue significantly increased medio-lateral postural oscillations in all subjects. Moreover, gymnasts did not present a better postural balance control than non-gymnasts. Based on electromyographic parameters, the former employed other neuromuscular control strategies to maintain their postures in single-legged quiet standing.

Gorjan et al. investigated the motion of the centre of mass while standing with external stabilization, without constraining the joint movements, using a method based on an inverted cart-pendulum system. Results showed inter-individual variability in postural responses during the external stabilization, with less than half of all subjects showing a stabilizing effect.

Kacem et al. examined the effect of neuromuscular fatigue on static and dynamic postural balance performance in female athletes during the premenstrual phase and the menstrual cycle. Results indicated that the disruptive effect of neuromuscular fatigue on static and dynamic postural control was more accentuated in the premenstrual phase than during the menstrual cycle.

Pojskic et al. investigated the influence of acute hypoxic exposure on balance ability in highly trained basketball players through repeated single-leg balance tests over a multiaxial tilting platform. The findings of this study highlighted how the acute effects of normobaric hypoxia on balance performance were modulated by the playing level. Indeed, elite players had better resistance to the adverse effects of normobaric hypoxia than their sub-elite counterparts.

Deng et al. showed the effect of hip joint angle on quadriceps recruitment pattern and stiffness in healthy individuals during isometric knee extensions.

Heredia-Elvar et al. focused on some of the most popular isometric core stability exercises. They provided new observational screening guidelines based on body alignment, postural sway, and pelvic acceleration thresholds calculated using a smartphone and its inbuilt accelerometer. These guidelines could help decide if a core stability exercise variation represents an adequate training intensity level for a given participant.

In a first review article, Zemkova noted that of the various studies that examined the effects of exercise on postural balance control, only a few were conducted under actual sport-specific conditions. Addressing this gap should have positive implications for developing specific exercise programs in those disciplines where success is linked to postural performance. In a second review article, Zemkova and Zapletalova highlighted that of the numerous studies that investigated the role of neuromuscular control of postural and core stability in sports performance, only a few demonstrated a relationships between body balance and stability of the core.

Ketterer et al. investigated whether continuous sensory conflicts induced by optic flow perturbations induced by virtual reality could challenge the postural system sustainably. The study results showed that sinusoidal optic flow perturbations reduced postural stability only in the first 5 s of the test, thus being not sufficiently suitable for balance training as they cannot trigger persisting sensory conflicts.

Kiers et al. presented a dynamic postural stability index based on the forces recorded during a ski-specific single-leg landing on a dynamometric platform after a forward double-leg drop jump from a box over a hurdle. The results suggested that this index could be a reliable and sensitive measure of dynamic postural control in youth skiers.

In conclusion, the Research Topic points to the need for further investigation in sports and daily-living contexts to understand the role of specific environmental conditions on postural balance performance. Given the specificity of balance concerning the external context, researchers are encouraged to develop new tests in addition to standardized laboratory tests. An interesting perspective is the employement of wearable technologies to enable the development of context-specific tests and overcome the lack of objectivity in the assessment of current ecological tests. An open Research Topic is how to effectively quantify the intensity of destabilizing exercises. Expanding this knowledge will support the development of tailored balance training programs to enhance athletic performance and reduce the risk of falls in daily life.

